# Immediate Orthodontic Repositioning of Traumatically Extruded Incisors in a Patient With Cleft Lip and Alveolus: A Case Report

**DOI:** 10.1155/crid/5705556

**Published:** 2026-02-26

**Authors:** I. Middeljans, M. A. R. Kuijpers, C. M. Suttorp

**Affiliations:** ^1^ Section of Orthodontics and Craniofacial Biology, Department of Dentistry, Radboud University Medical Center, Nijmegen, the Netherlands, radboudumc.nl

**Keywords:** cleft lip and alveolus, dental splinting, dental trauma, dental trauma guidelines, extrusive luxation, orthodontic repositioning, pulp necrosis

## Abstract

This case report describes a 15‐year‐old boy born with a cleft lip and alveolus who sustained severe extrusive and lateral luxation trauma to multiple incisors during the final phase of orthodontic treatment. The displacement caused incisal interference and prevented normal jaw closure. Deformed beta titanium wires were removed, loosened brackets were rebonded, and immediate orthodontic repositioning was initiated using light forces with 0.014‐inch CuNiTi round wires combined with manual repositioning. This approach resolved the incisal interference and restored functional occlusion within 1 week, with complete incisal alignment achieved after 3 months. During follow‐up, several affected teeth required endodontic treatment due to pulp necrosis. The patient’s cleft‐related dental anomalies prolonged the overall treatment process. This case highlights the potential benefit of immediate orthodontic repositioning after extrusive dental trauma, a treatment option currently underrepresented in existing dental trauma guidelines.

**Summary**

• Light orthodontic forces restored jaw closure and occlusion within 1 week following dental trauma.
• Cleft‐related dental anomalies prolonged the overall treatment.
• This report identifies a gap in dental trauma guidelines, which do not recommend immediate orthodontic repositioning of extrusively luxated teeth.
• The case supports active orthodontic repositioning as a rapid, functional alternative to passive splinting in certain cases.

## 1. Introduction

Cleft lip and/or palate (CLP) is a common congenital craniofacial anomaly, affecting ~1 in 700 newborns and characterized by substantial phenotypic variation [1]. The cleft can involve the upper lip, alveolus, and/or palate. Depending on severity, individuals with CLP require long‐term, multidisciplinary care from infancy through adulthood, including surgical and orthodontic interventions [2]. Traumatic dental injuries are common during childhood and early adolescence [3]. Since patients with CLP often undergo orthodontic treatment at multiple stages of rehabilitation [4, 5], the risk of dental trauma during orthodontic care is relatively high. Despite this, case reports documenting such incidents in CLP patients remain scarce.

Individuals with CLP have an increased prevalence of dental anomalies, including variations in tooth size, shape, number, and development [6]. The maxillary lateral incisor on the cleft side is the most commonly missing tooth, absent in ~39.1% of cases [7]. Patients with CLP often present with shortened roots affecting the maxillary incisors, canines, and other teeth [8]. In individuals with complete unilateral cleft lip and palate who are treated with fixed orthodontic appliances, a higher incidence of external apical root resorption has been reported in the maxillary anterior teeth on the cleft side [9]. Additionally, supernumerary canals may complicate endodontic treatment [10]. Increased susceptibility to dental caries and periodontitis further highlights the need for early, preventive, and individualized dental care for CLP patients [11].

Extrusive luxation is a type of dental trauma in which a tooth is axially displaced from its alveolar socket, resulting in disruption of the periodontal ligament attachment and rupture of the apical neurovascular bundle, leading to increased tooth mobility. The affected tooth is often appears elongated and is displaced palatally or lingually, causing incisal interference that obstructs jaw closure and disrupts occlusion [12]. The International Association of Dental Traumatology (IADT) recommends prompt repositioning of luxated permanent teeth, either manually or with forceps, followed by stabilization using a passive flexible splint [13, 14]. However, immediate repositioning may be impeded by alveolar bone deformation or blood clots [15, 16], potentially necessitating immediate orthodontic repositioning to resolve incisal interference and restore functional jaw closure.

Immediate orthodontic repositioning of extruded teeth is not included in the IADT Dental Traumatology Guidelines [13], the Dental Trauma Guide [12], or the European Society of Endodontology guidelines [17]. Although dental trauma is widely reported, there is limited evidence on the role of immediate orthodontic repositioning. This case report highlights the potential benefits of using immediate orthodontic repositioning to restore incisal relationships and occlusion.

## 2. Case Presentation

### 2.1. Diagnosis and Etiology

#### 2.1.1. Phase I: Before the Dental Trauma Event

**Figure 1:**
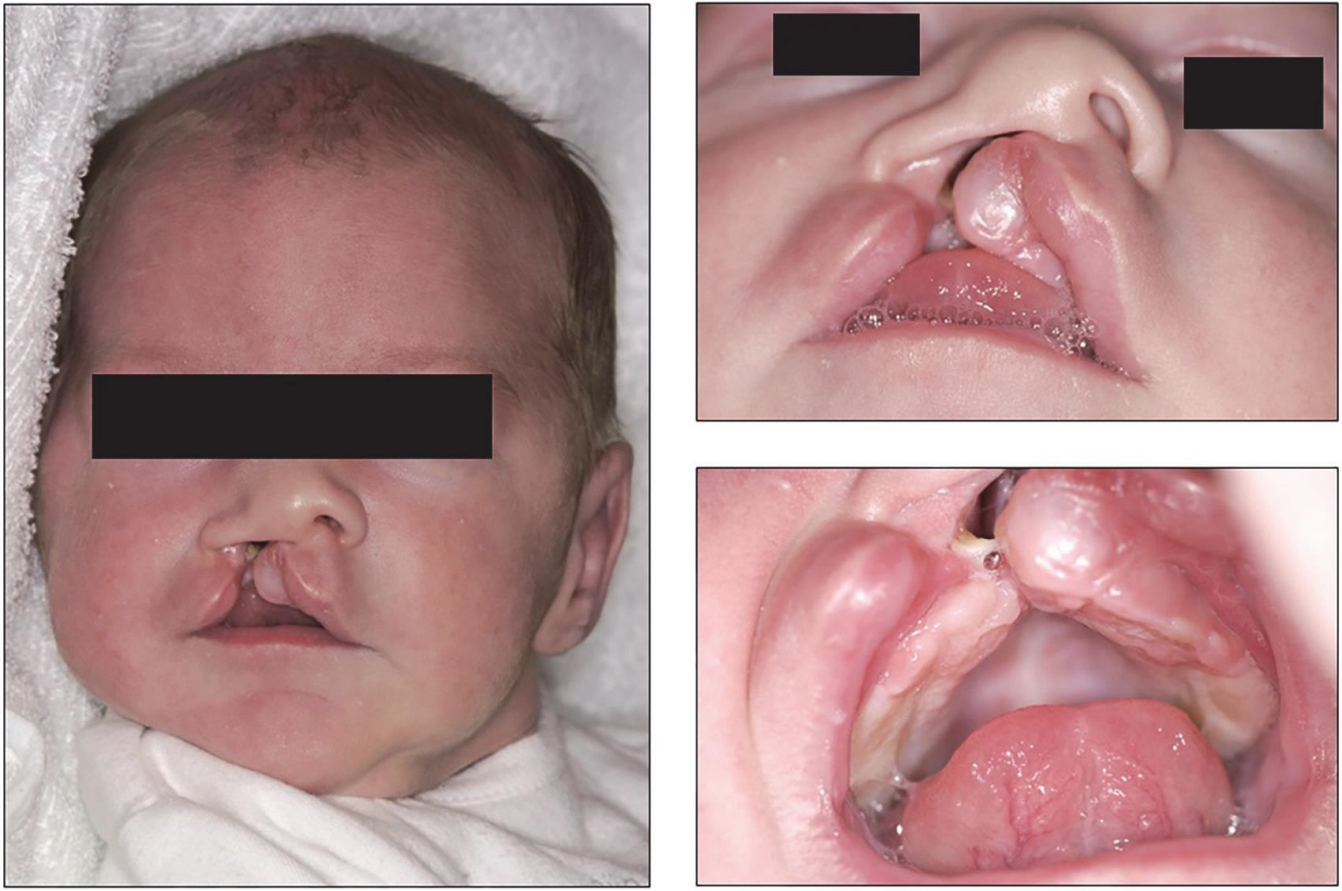
Extraoral and intraoral photographs of the patient at 3 weeks of age.

**Figure 2:**
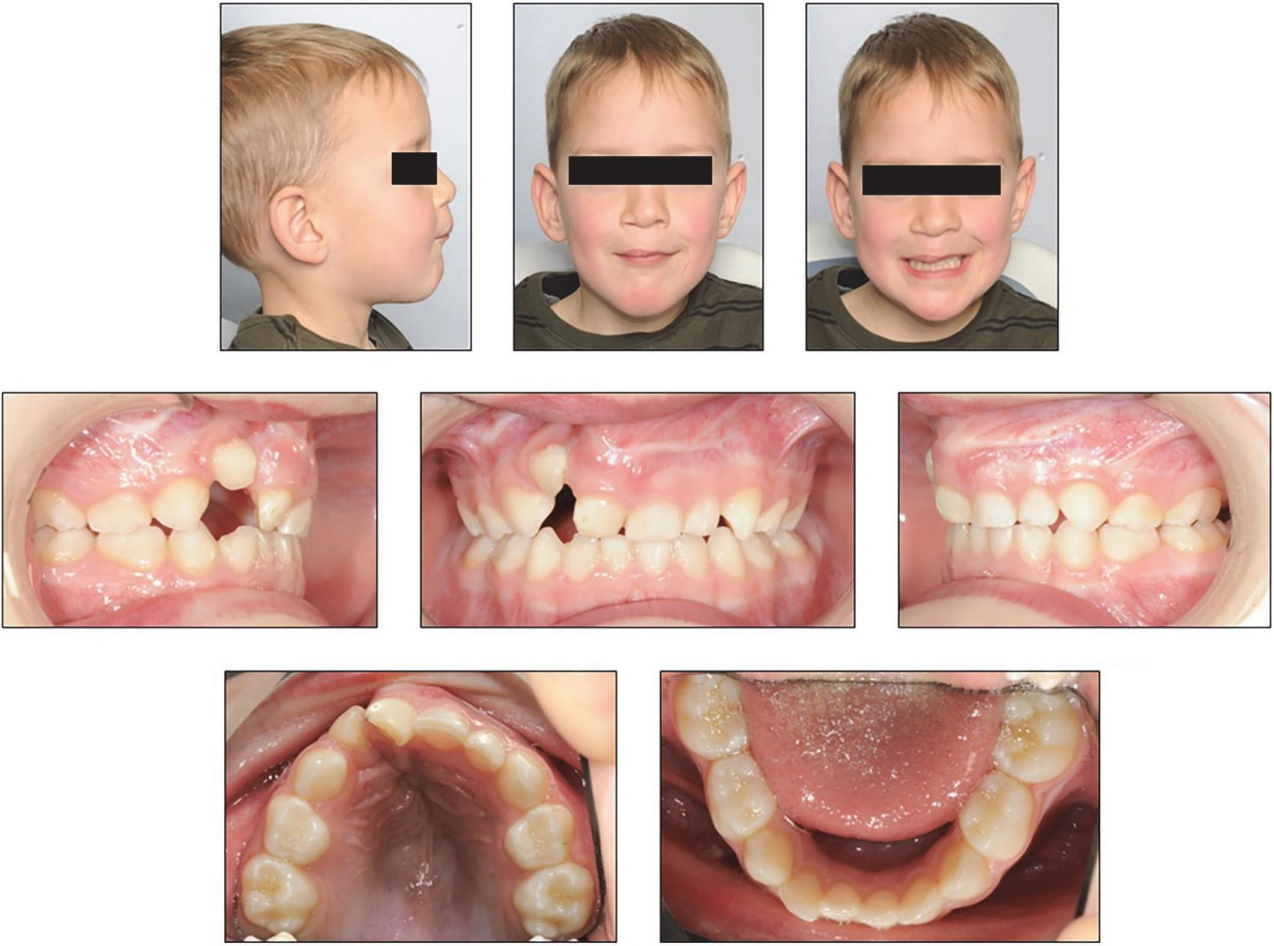
Extraoral and intraoral photographs of the patient at 4 years and 11 months of age.

**Figure 3:**
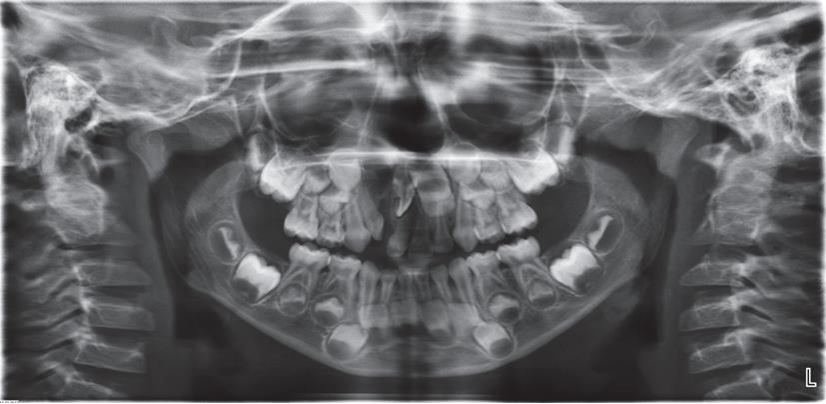
Panoramic radiograph of the patient at 4 years and 11 months of age.

The patient was born with a complete unilateral cleft lip and alveolus on the right side and was referred to the multidisciplinary cleft team shortly after birth (Figure 1). Growth and development were routinely monitored. At 4 years and 11 months, intraoral and extraoral records were obtained (Figure 2). The maxillary right primary lateral incisor was missing, and panoramic radiography confirmed agenesis of the maxillary right permanent lateral incisor (Figure 3).

**Figure 4:**
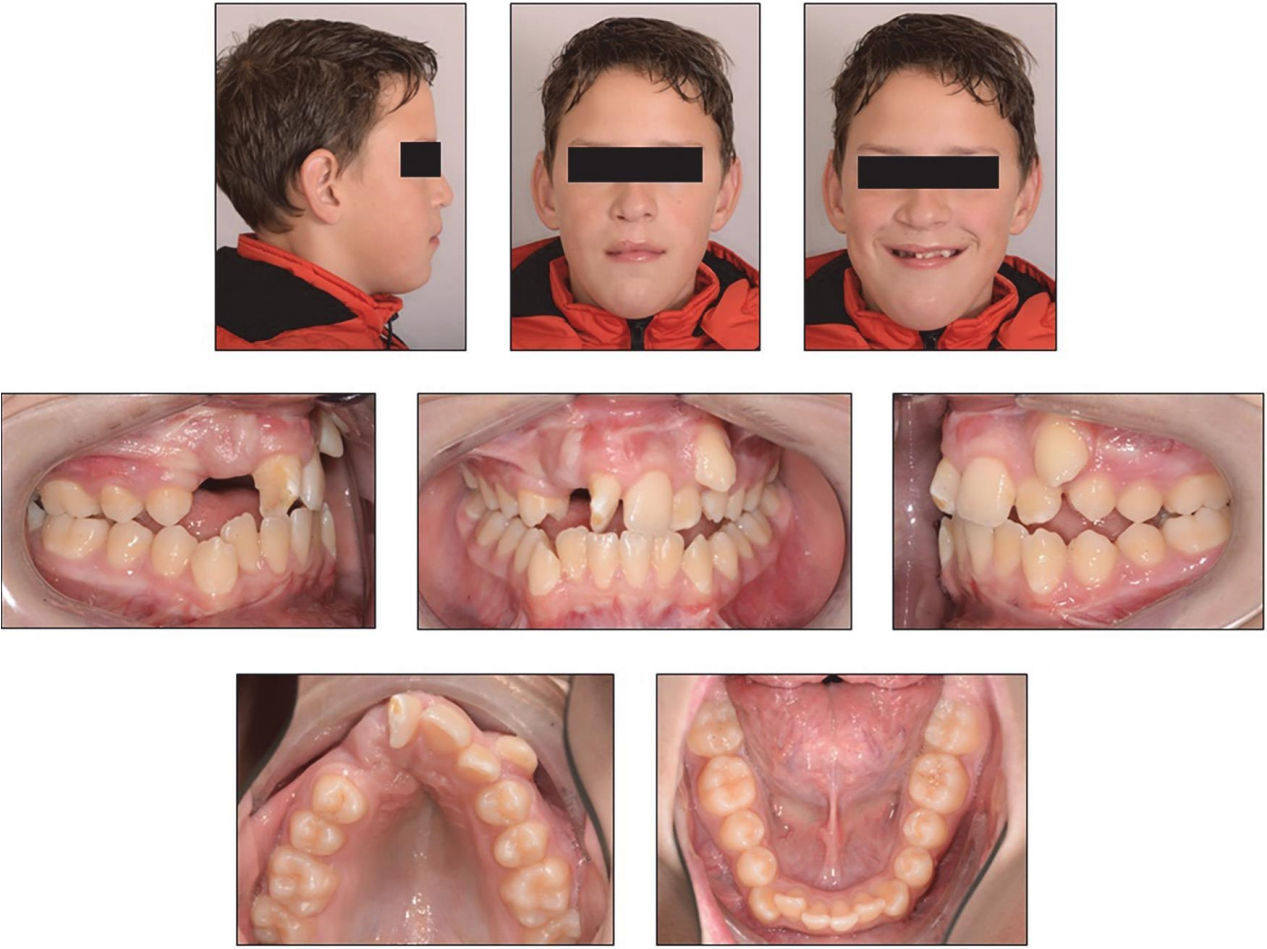
Extraoral and intraoral photographs of the patient at 13 years and 6 months of age.

At 10 years and 4 months, alveolar bone grafting was performed using autogenous chin bone, without prior orthodontic treatment. At 13 years and 6 months, follow‐up records were taken to evaluate dental development (Figure 4). The patient exhibited a half‐cusp Class II molar relationship on the right and a quarter‐cusp Class II on the left, with 3 mm overjet and 5 mm overbite. The maxillary right central incisor displayed ~45° mesiopalatal rotation, enamel dysplasia, and dentin discoloration. The maxillary right canine was palpable buccally along the alveolar ridge. Significant crowding of the lower anterior teeth was present due to limited space.

The treatment plan aimed to achieve aligned arches with stable occlusion and incisal relationships. Extraction of the maxillary left first premolar was planned to create space for the left canine, while both mandibular first premolars were extracted to resolve lower anterior crowding. Full fixed appliances were planned, with a composite veneer on the maxillary right central incisor for esthetic improvement.

The premolars were extracted by the dentist, followed by bonding of full fixed appliances in the maxillary arch, and 5 months later in the mandibular arch. Passive self‐ligating brackets with 0.022‐inch × 0.028‐inch slots (Damon system, Ormco, Glendora, CA) were used. Initial alignment and leveling started with a 0.013‐inch CuNiTi round wire, followed by a 0.018‐inch CuNiTi wire. Rectangular wires were applied sequentially: 0.014‐inch × 0.025‐inch CuNiTi, 0.018‐inch × 0.025‐inch CuNiTi, 0.017‐inch × 0.025‐inch beta titanium (TMA), and 0.019‐inch × 0.025‐inch TMA (Ormco, Glendora, CA). After 23 months of active treatment, the finishing phase was reached, during which final corrections were made to achieve optimal alignment and occlusion.

#### 2.1.2. Phase II: After the Dental Trauma Event

**Figure 5:**
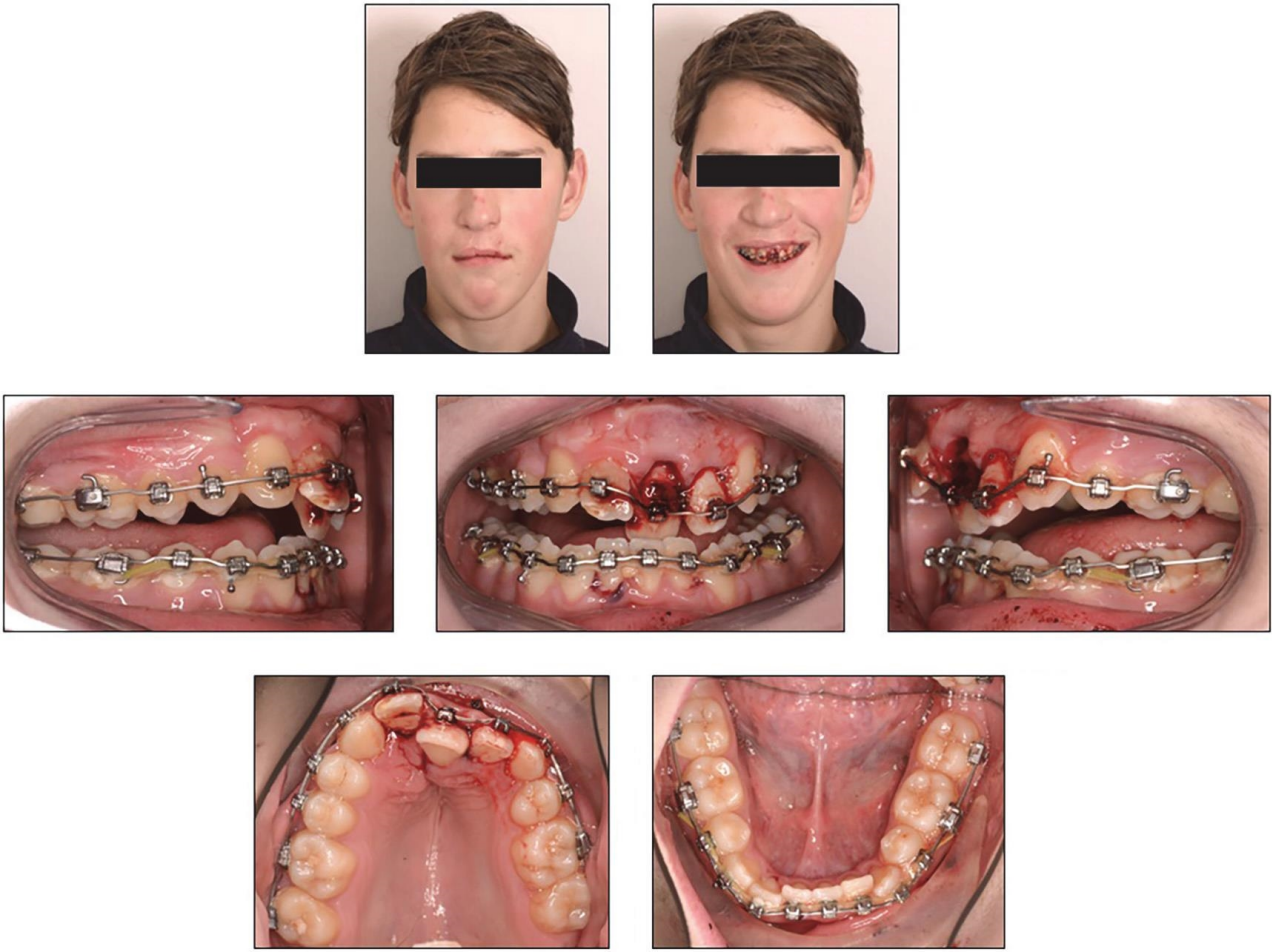
Extraoral and intraoral photographs of the patient at 15 years and 8 months of age, taken on the day of the dental trauma.

After 24 months of orthodontic treatment, the patient reported by telephone that he had sustained dental trauma after falling from his bike and hitting the sidewalk. Several upper and lower anterior teeth were severely displaced, prompting an emergency appointment the same afternoon. Clinical examination revealed severe extrusion and lateral luxation of the maxillary left central and lateral incisors toward the palatal side, while the maxillary right central incisor showed minor extrusion. In the mandibular arch, the left central and lateral incisors and the right central incisor exhibited extrusive and lateral luxation toward the lingual side. The trauma caused an anterior end‐to‐end incisal relationship, resulting in incisal interference that prevented normal jaw closure and occlusion. The 0.019‐inch × 0.025‐inch TMA rectangular wires in both arches were heavily deformed due to the trauma (Figure 5). No other symptoms were reported.

**Figure 6:**
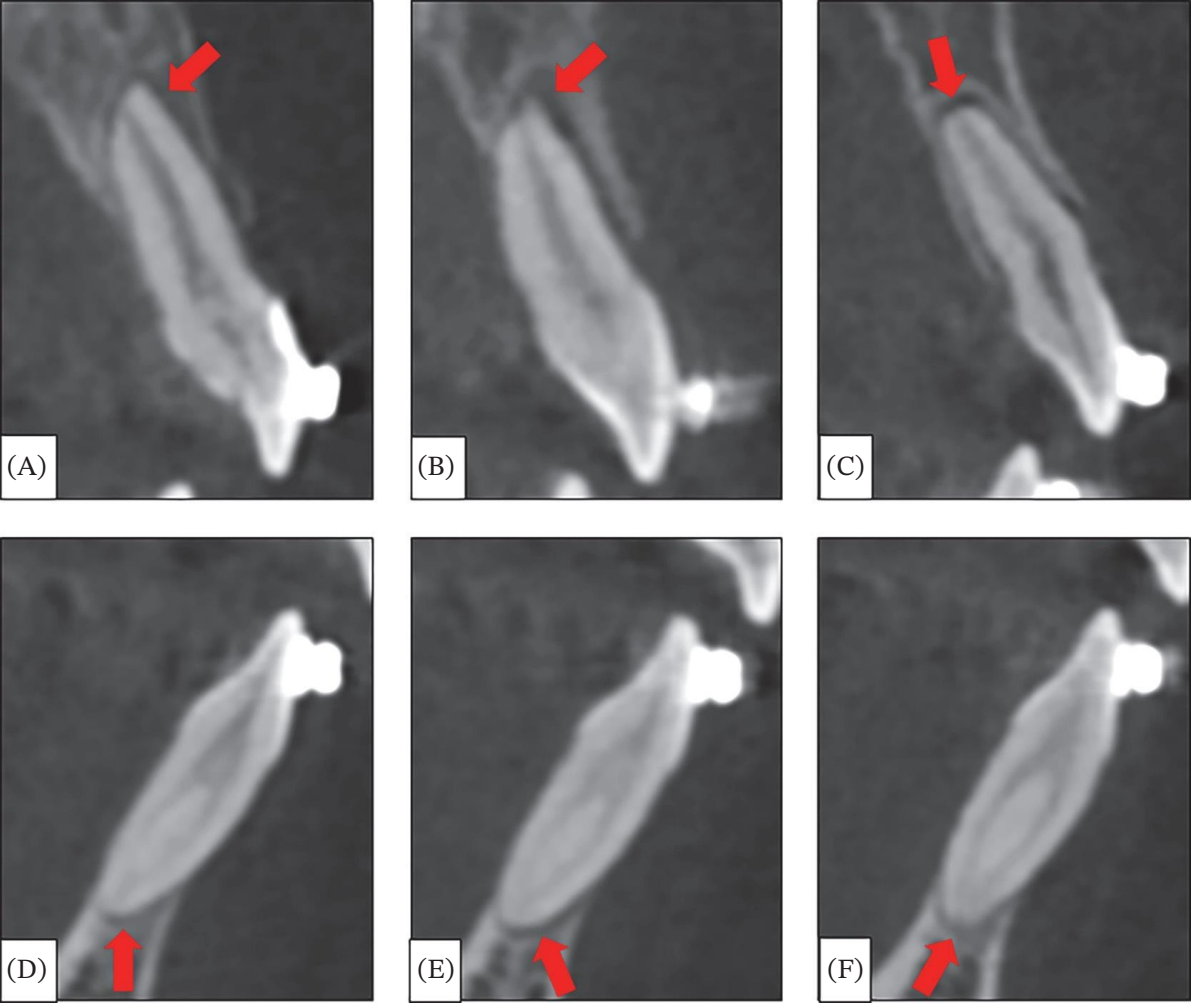
Sagittal dental CBCT sections of the luxated teeth in the maxilla and mandible: (A) maxillary right central incisor, (B) maxillary left central incisor, (C) maxillary left lateral incisor, (D) mandibular right central incisor, (E) mandibular left central incisor, and (F) mandibular left lateral incisor. Red arrows indicate areas with an enlarged apical periodontal ligament space.

The deformed orthodontic wires were removed, and loosened brackets on the displaced anterior teeth were rebonded. A dental cone‐beam computed tomography (CBCT) scan of the maxillary and mandibular regions (voxel size 0.3 mm) was performed to rule out alveolar bone and root fractures. CBCT revealed enlarged periodontal ligament spaces at the apex of the maxillary right central incisor (Figure 6A), the maxillary left central and lateral incisors (Figure 6B,C), the mandibular right central incisor (Figure 6D), and the mandibular left central and lateral incisors (Figure 6E,F), indicating axial displacement of the roots. No alveolar bone or root fractures were detected.

**Figure 7:**
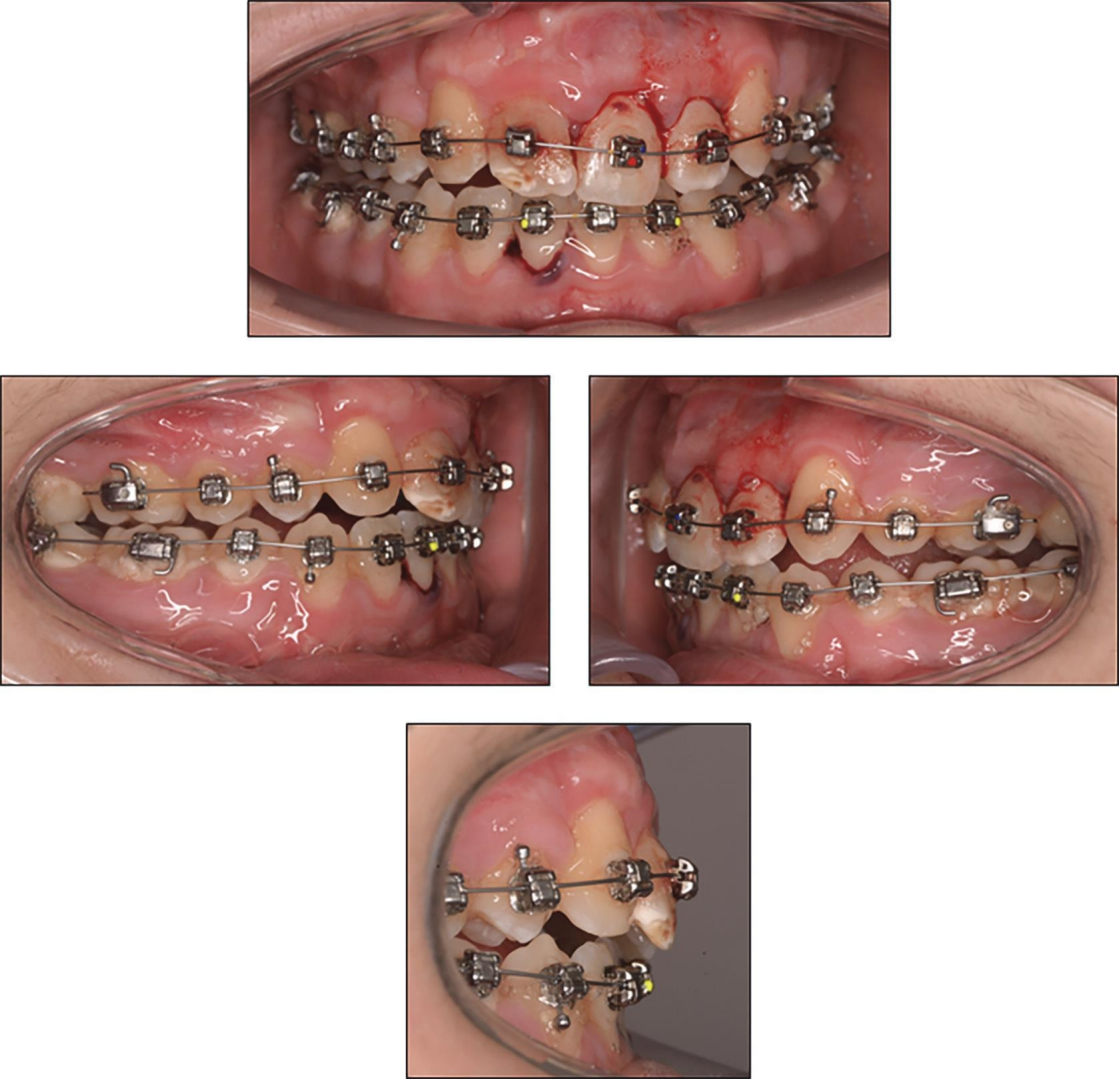
Intraoral photographs taken immediately after emergency treatment, following manual repositioning and placement of a 0.014‐inch CuNiTi round wire in both arches.

Light orthodontic forces were applied to both arches using a 0.014‐inch CuNiTi round wire, combined with gentle manual repositioning, to correct incisal interference and restore normal jaw closure (Figure 7). Due to the severity of the displacement, pulp vitality loss was suspected in some teeth. The patient’s dentist agreed to regularly monitor anterior tooth sensitivity using ethyl chloride testing to assess vitality.

### 2.2. Treatment Alternatives

The IADT Dental Traumatology Guidelines indicate that occlusal interferences are common in displaced teeth, but the recommended treatment is limited to repositioning followed by passive splinting with a flexible wire [13, 14]. In this case, however, due to the goal of fully restoring the incisal relationship, treatment was not limited to passive splinting. Instead, the teeth were repositioned, and active orthodontic treatment using light forces was initiated immediately. Passive splinting alone was considered insufficient to correct the functional disturbance. Importantly, immediate active orthodontic repositioning of traumatically extruded teeth is not mentioned as an option in the IADT guidelines, highlighting a notable gap. Active orthodontic repositioning can more effectively restore occlusion and prevent prolonged functional impairment. Therefore, in this case, treatment deliberately deviated from the guidelines to more rapidly restore normal jaw function and occlusion.

### 2.3. Treatment Progress

**Figure 8:**
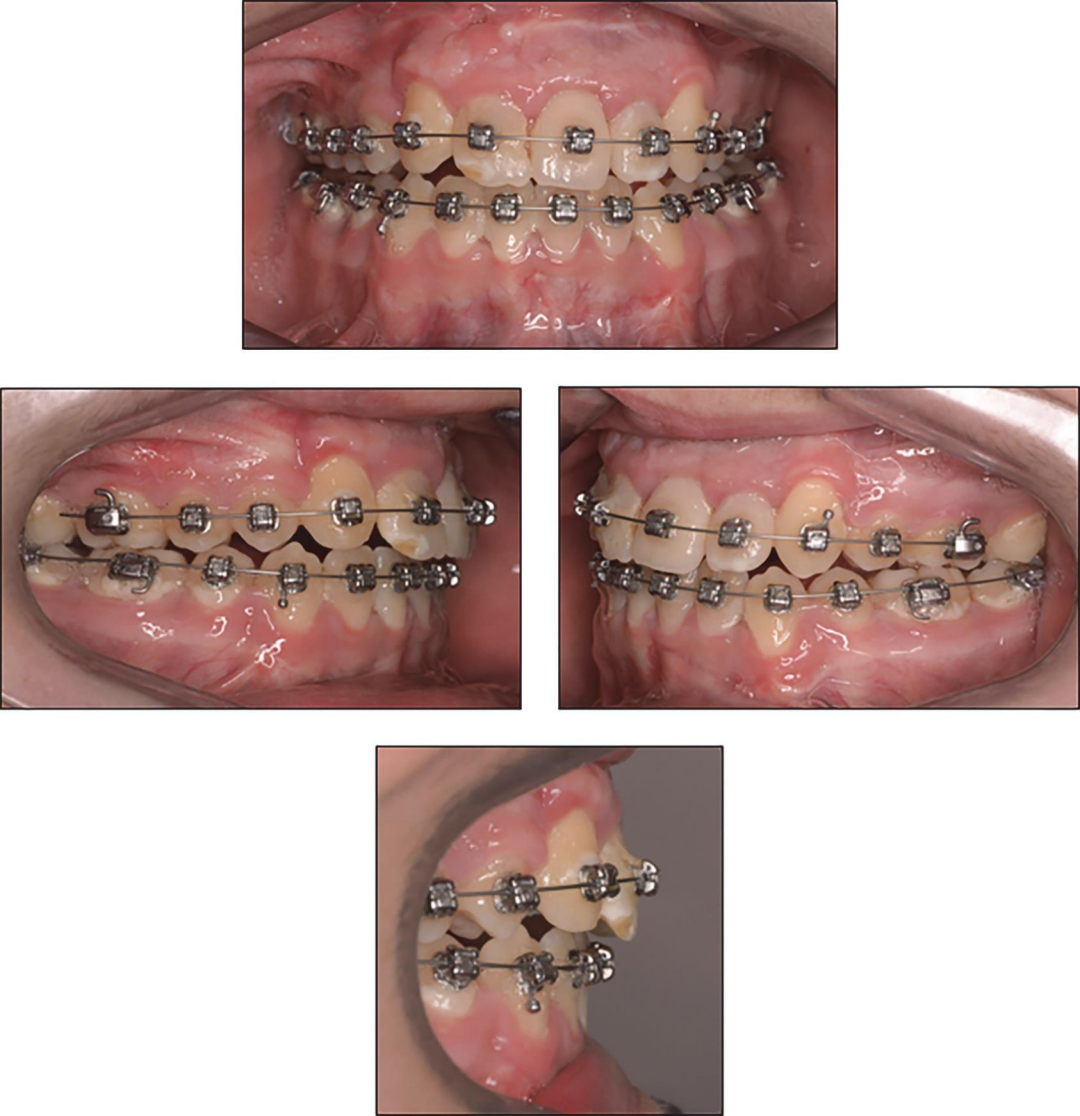
Intraoral photographs of the patient with 0.014‐inch copper–nickel–titanium round wires in place, taken 2 weeks after the trauma.

Within 1 week after trauma, the patient regained normal jaw closure, and after 2 weeks, orthodontic alignment had largely restored the anterior incisal relationship (Figure 8).

**Figure 9:**
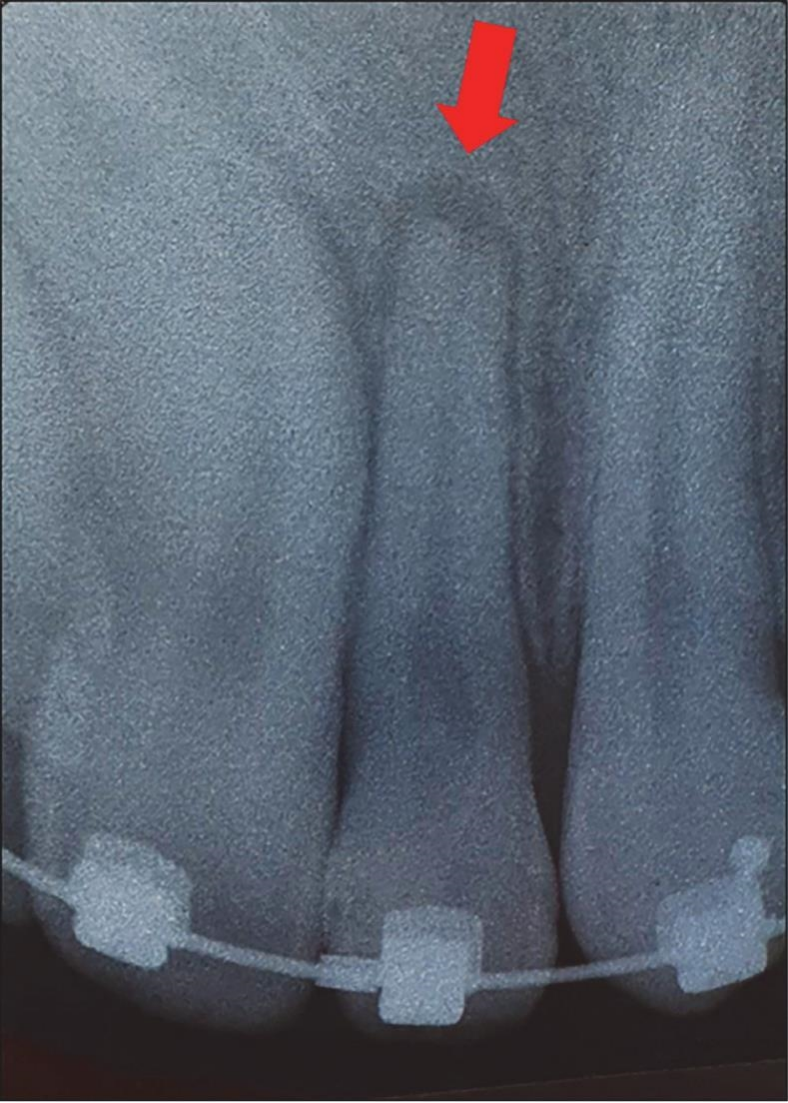
Periapical radiograph of the maxillary left lateral incisor, taken 8 weeks after the trauma. Red arrows indicate the apical radiolucency.

At 8 weeks posttrauma, the maxillary left lateral incisor showed new gray discoloration, suggesting pulpal necrosis. A periapical radiograph revealed apical radiolucency indicative of apical periodontitis (Figure 9). The patient was referred to his dentist, who performed root canal treatment.

**Figure 10:**
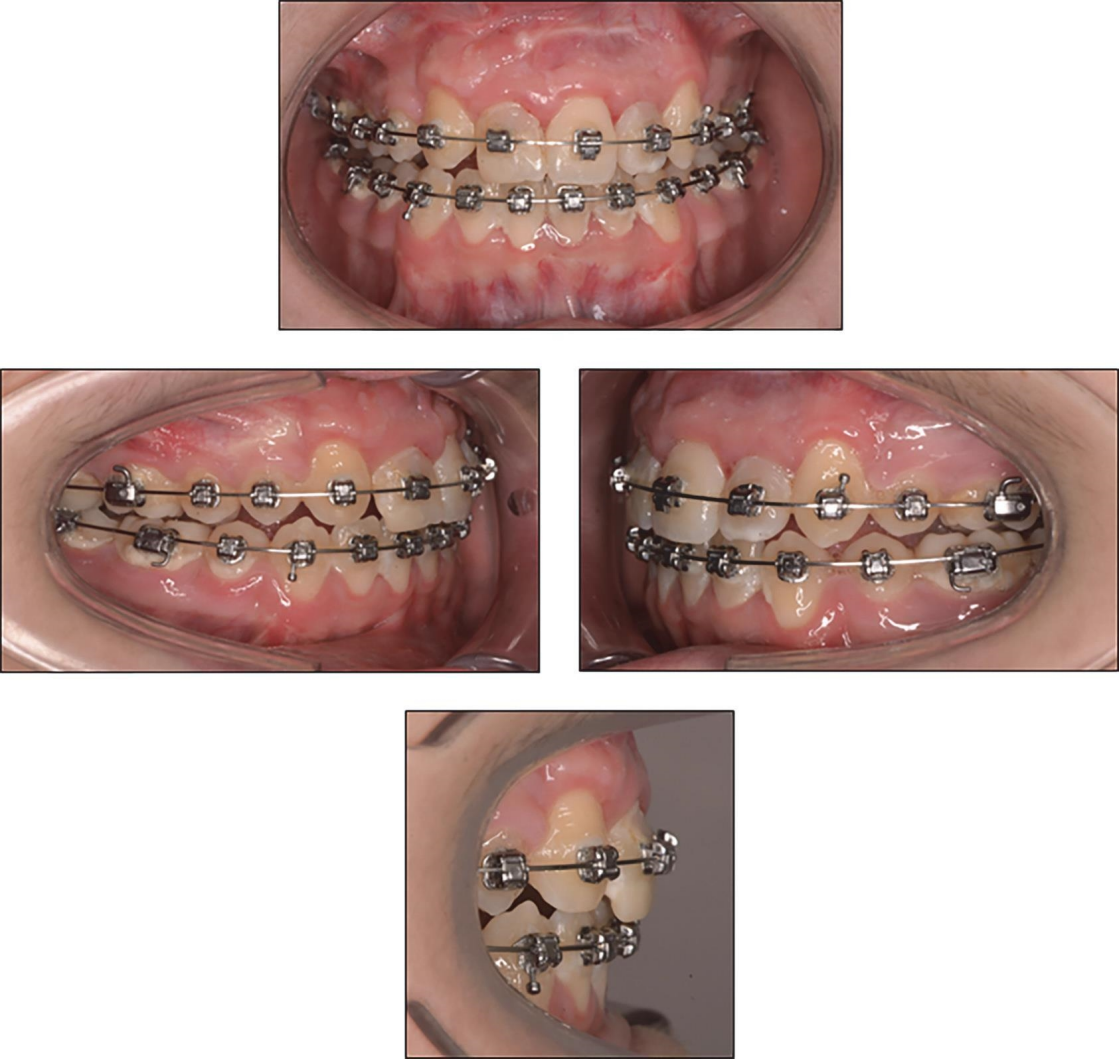
Intraoral photographs of the patient taken 3 months after the trauma, following placement of 0.017‐inch × 0.025‐inch SS wires.

Over 3 months, both arches were sequentially leveled and aligned using 0.014‐inch × 0.025‐inch and 0.018‐inch × 0.025‐inch CuNiTi wires. Subsequently, 0.017‐inch × 0.025‐inch stainless steel (SS) wires were placed in both arches (Figure 10). Class II elastic traction (3.5 oz, ¼ inch) was applied to optimize the sagittal relationship between maxillary and mandibular anterior teeth.

During orthodontic treatment, the dentist performed additional root canal treatments on the mandibular left central and lateral incisors. Ten months after the trauma, the patient was satisfied with the outcome and requested removal of the fixed appliances.

### 2.4. Treatment Results

**Figure 11:**
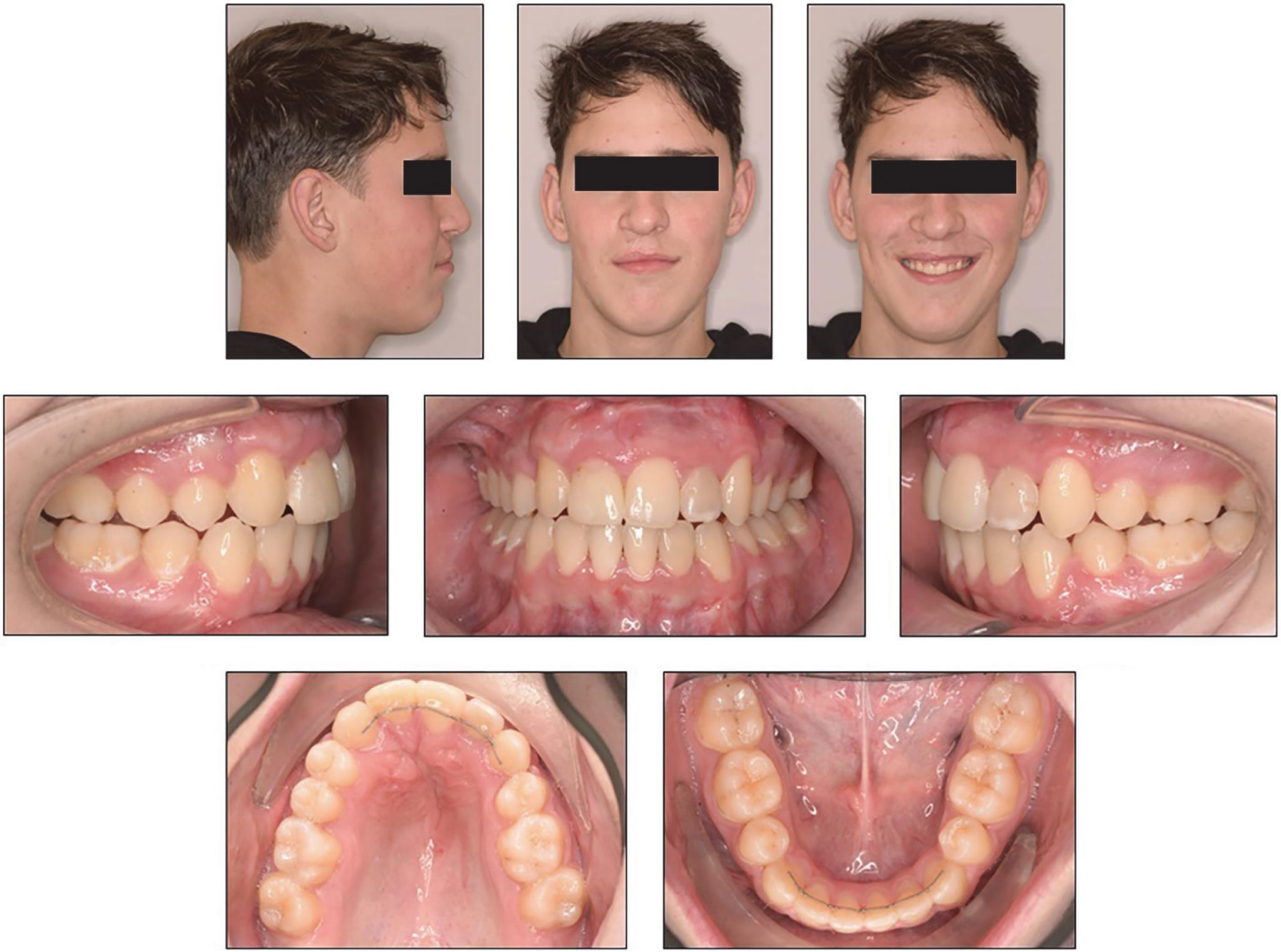
Extraoral and intraoral photographs of the patient at the age of 16 years and 8 months.

The fixed orthodontic appliances were removed, and the maxillary right central incisor was restored with a composite veneer. A bonded canine‐to‐canine retainer (C–C bar) was placed in both the upper and lower arches, and facial and intraoral photographs were taken (Figure 11). Ideal overjet and overbite (2 mm each) were achieved. A removable Hawley retainer was provided in the upper arch for overnight use to maintain arch form and prevent relapse.

**Figure 12:**
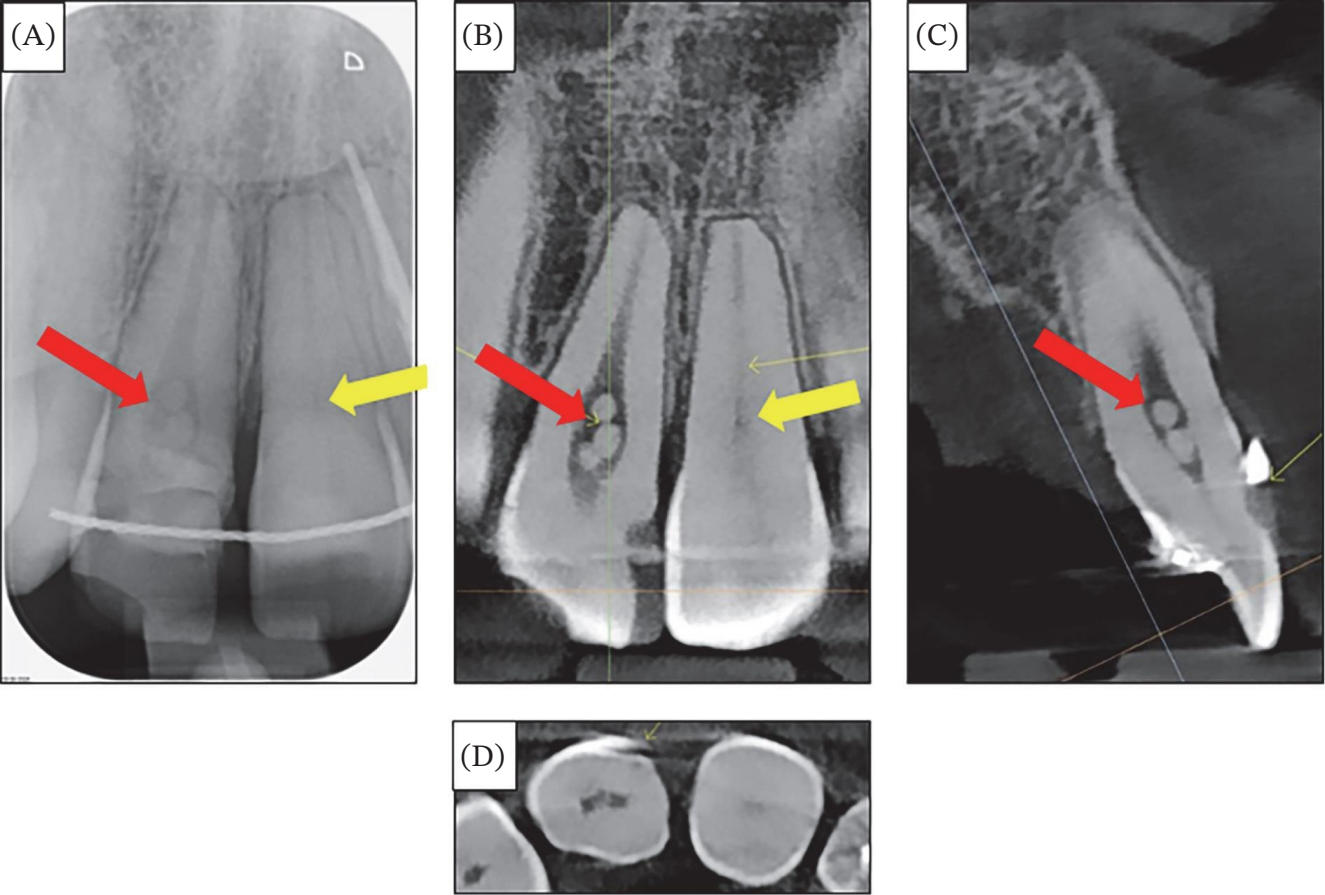
Radiographic images of the maxillary right central incisor taken to evaluate the presence of external cervical resorption. (A) Periapical radiograph of the maxillary right central incisor. (B) Coronal section of the cone‐beam CT through the maxillary central incisors. (C) Sagittal section of the cone‐beam CT through the maxillary right central incisor. (D) Transversal section of the cone‐beam CT through the maxillary central incisors. The red arrow indicates spherical calcifications in the pulp chamber, and the yellow arrow indicates obliteration of the pulp canal.

At the retention check, 2 months after debonding, clinical examination suggested possible external cervical resorption in the maxillary right central incisor, indicated by a subgingival ledge and pink crown discoloration. A periapical radiograph (Figure 12A) showed vague radiolucency. The patient was referred to an endodontist, and a dental CBCT scan was performed (Figure 12B–D). No external cervical resorption was observed upon further investigation. The CBCT revealed a slight overhang of the composite veneer on the buccal surface and spherical calcifications in the pulp chamber, which did not require treatment, and pulp chamber obliteration in the maxillary left central incisor.

**Figure 13:**
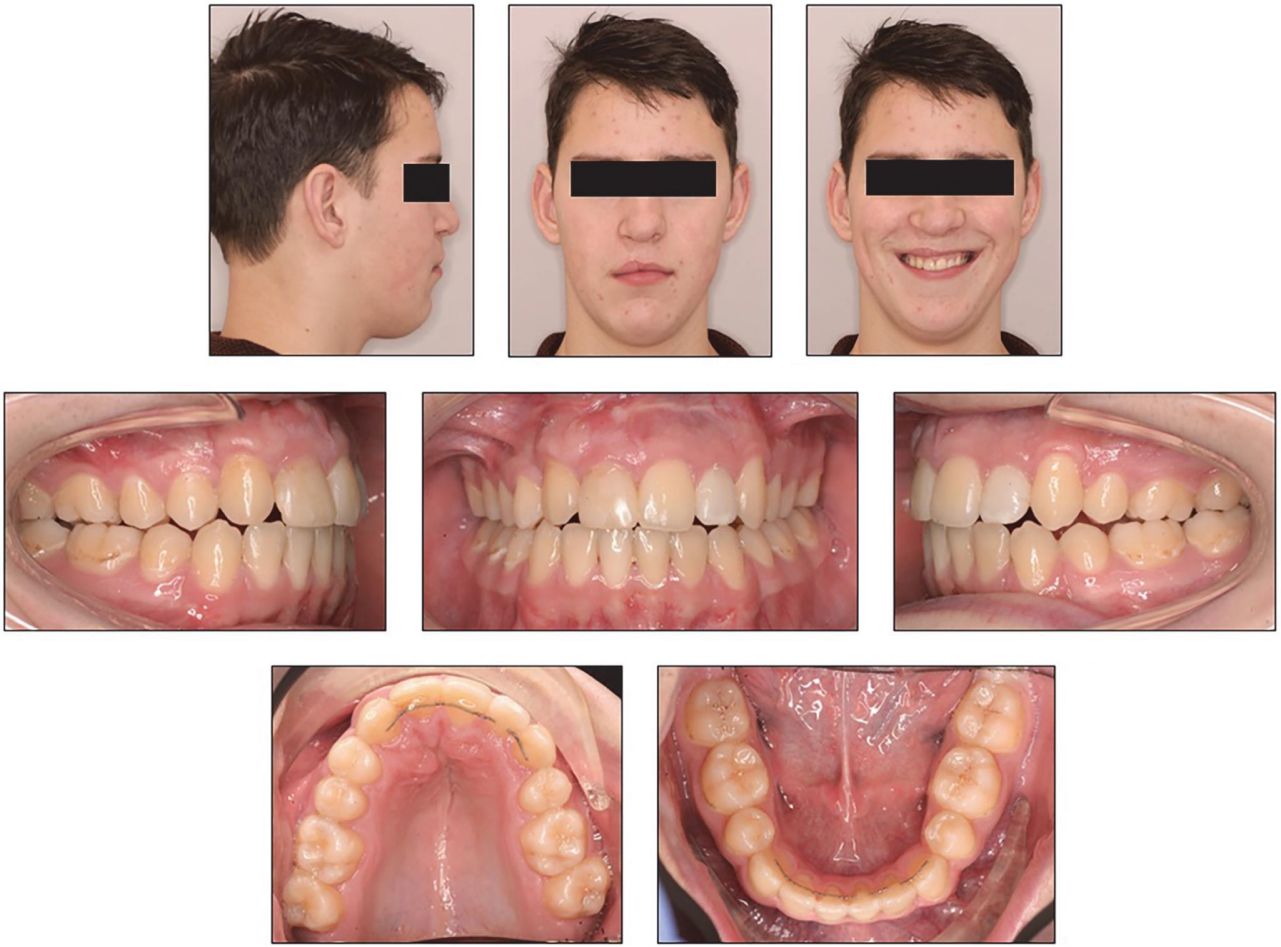
Extraoral and intraoral photographs of the patient, taken at the age of 17 years and 10 months, 1 year and 3 months after treatment.

About 1 year and 3 months after treatment, occlusion and arch forms remained stable. Bonded canine‐to‐canine retainers were intact (Figure 13), and the patient consistently used the Hawley retainer overnight.

**Figure 14:**
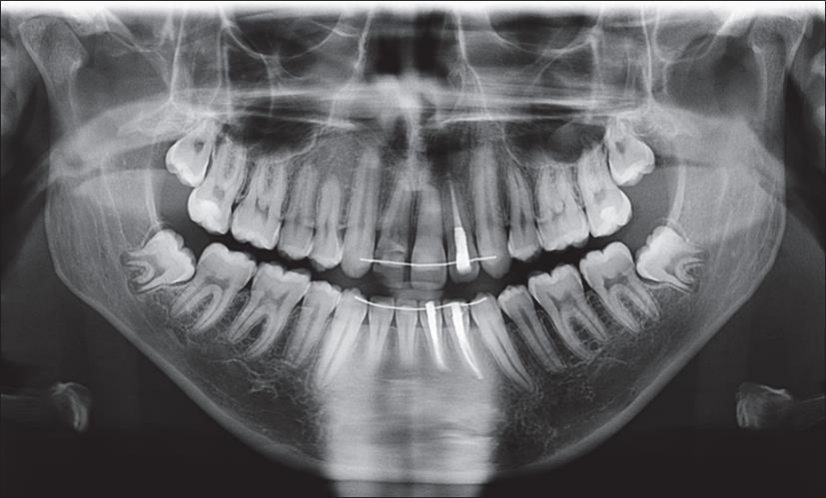
A panoramic radiograph was taken at the age of 17 years and 10 months.

A panoramic radiograph (Figure 14) confirmed no new pathology or complications in the previously endodontically treated mandibular left central and lateral incisors.

The patient and his parents were satisfied with the treatment outcomes. Restoring normal jaw closure and aligning the anterior teeth resolved functional issues, such as difficulties with biting and chewing, while improving daily comfort. The patient was also pleased with the esthetic result, noting a significant improvement in the appearance of his smile. During the retention phase, both the functional and esthetic outcomes were maintained, and the patient remained satisfied, with the treatment positively impacting his daily life and confidence.

## 3. Discussion

Individuals with CLP exhibit a higher prevalence of dental anomalies compared to non‐CLP individuals. Common defects include enamel hypoplasia, dentin dysplasia, microdontia, peg‐shaped teeth, taurodontism, and lateral incisor agenesis [18–20]. Enamel discoloration, particularly in maxillary anterior teeth on the cleft side, suggests a direct effect of the cleft on tooth development [19, 21]. Anatomical canal variations, usually rare in anterior teeth, are more frequent in CLP patients and may go unnoticed [22]. The etiology of these anomalies is multifactorial: surgical trauma from procedures like periosteoplasty can disrupt blood supply, contributing to developmental defects, and insufficient early orthopedic treatment of the maxillary segments further increases risk [20]. In this case, agenesis of the maxillary right lateral incisor was observed, and the maxillary right central incisor showed enamel dysplasia and dentin discoloration with an enlarged pulp chamber, consistent with dental anomalies commonly reported in individuals with CLP.

Several studies have examined the prevalence and characteristics of traumatic dental injuries in both CLP and non‐CLP populations. da Silva et al. [23] reported that rates of avulsion, luxation, and intrusion in individuals with CLP were similar to noncleft individuals. No other studies directly compared dental injury prevalence between these groups. Berger et al. [24] analyzed cases requiring splinting, finding 40 individuals affected, most with multiple injured teeth: 21 lateral luxations, 19 extrusions, 2 intrusions, and 13 avulsions. The main causes were sports (48%), accidental falls (41%), and violence (11%). Amilcar et al. [25] reviewed 366 records, identifying 166 patients (350 teeth) with luxation injuries, more common in males (*n* = 102) than females (*n* = 64). Extrusive luxation was most frequent (99 patients, 208 teeth) and often required endodontic treatment, while lateral luxation was strongly linked to traffic accidents. In the present case, the combination of luxation trauma, male gender, and a fall from the bicycle aligns with these reported patterns.

Extrusive luxation injuries have been studied for their long‐term effects on pulp and periodontal healing. Hermann et al. [26] reported that, in 149 patients with extrusive luxation trauma, mature teeth had a low risk of complications: 17.5% marginal bone loss, 15.6% repair‐related resorption, 5.1% infection‐related resorption, and 0% ankylosis. Coste et al. [27] examined 224 patients with 427 luxated permanent teeth, finding pulp necrosis in 120 teeth (28.1%) and pulp canal obliteration in 55 teeth (12.9%). In the present case, the pulp of the maxillary left lateral incisor and mandibular left central and lateral incisors became necrotic following extrusive luxation, and pulp canal obliteration was observed in the maxillary left central incisor. Radiographically, extrusively luxated teeth often show enlarged periodontal ligament spaces [28], as seen in the CBCT images. These outcomes—pulp necrosis, pulp canal obliteration, and enlarged periodontal ligament space—are consistent with existing literature on extrusive luxation injuries.

Several studies have investigated pulp stones in different patient populations, examining associations with trauma, cleft conditions, and orthodontic treatment. Zahran and Alamoudi [29] found that 97.6% of teeth with pulp stones had no history of trauma. al‐Hadi Hamasha and Darwazeh [30] reported a similar prevalence of pulp stones in CLP and noncleft individuals. Babanouri et al. [31] analyzed 100 patients with fixed orthodontic appliances, finding pulp stones in 17% before treatment and 35% after, suggesting orthodontic treatment may contribute to their formation. In this case, the pulp stone observed in the maxillary right central incisor is likely related to orthodontic treatment rather than trauma or the cleft condition, consistent with the literature.

AlMogbel et al. [32] reported that orthodontic tooth movement may start immediately after endodontic treatment. However, they recommend a 3‐month follow‐up before orthodontic movement in traumatized teeth after extrusive luxation to reduce root resorption risk. In this case, the maxillary left lateral incisor received endodontic treatment after orthodontic repositioning and correction of incisal interference. The mandibular left central and lateral incisors underwent root canal treatment near the end of orthodontic therapy. Despite guideline recommendations, orthodontic treatment was continued because only minimal movement was required. After 15 months of follow‐up, no trauma‐related root resorption was observed.

Dental splints are primarily used to support, protect, and stabilize teeth that have become mobile following trauma, surgery, or replantation. Flexible splints allow limited physiological movement while maintaining tooth position, which promotes periodontal healing [33]. In contrast, rigid splints, once widely used, have been linked to adverse outcomes such as pulp necrosis and ankylosis‐related root resorption [34]. The transition toward flexible splinting has markedly reduced the incidence of ankylosis, highlighting the importance of controlled functional mobility during healing [35]. Common splinting materials for luxated teeth include SS wire combined with composite resin and fiber‐reinforced splints [36].

According to the IADT Dental Traumatology Guidelines [13], the Dental Trauma Guide [12], and the ESE guidelines [17], management typically involves a passive splinting “rest” period with a flexible splint to allow initial healing. However, in the present case, the need for rapid restoration of functional occlusion justified the immediate initiation of active orthodontic treatment. Light orthodontic forces were applied using 0.014‐inch copper–nickel–titanium round wires. CBCT imaging revealed an enlarged apical periodontal ligament space, indicating displacement of the teeth from the alveolar socket. With the application of low orthodontic forces, the teeth were repositioned into the socket within 2 weeks, and no ankylosis was observed.

Orthodontic repositioning of traumatically extruded teeth is not routinely included in current dental trauma guidelines. However, several case reports support its clinical effectiveness. Owtad et al. [37] demonstrated that light orthodontic forces applied shortly after trauma to maxillary incisors successfully resolved incisal interference. Elbay et al. [38] reported effective orthodontic repositioning of a severely traumatized maxillary central incisor within 3 months. Similarly, Aarts and Suttorp [39] described the repositioning of the maxillary right lateral incisor and canine, achieving normal occlusion within 3 weeks. These findings suggest that early orthodontic intervention can provide both functional and esthetic benefits in selected trauma cases.

More recently, orthodontic repositioning has gained recognition as an alternative to manual repositioning or surgical fixation, particularly for extrusive and lateral luxation injuries. Spinas et al. [40] introduced delayed orthodontic repositioning, a gradual technique applying light, biologically acceptable forces over 6–8 weeks, resulting in minimal patient discomfort. Their 2024 review further supported this approach and recommended it as a viable alternative to conventional treatment methods [41]. Although orthodontic repositioning is more commonly used for dental intrusion injuries, its application in extrusive and lateral luxation cases remains limited [12]. Nevertheless, available clinical evidence, including the present case, indicates that early application of light orthodontic forces can effectively restore occlusion and support periodontal healing.

## 4. Conclusion

This case illustrates the clinical impact of extrusive luxation in a patient with cleft‐related dental anomalies. The successful early application of light orthodontic forces demonstrates orthodontic repositioning as a controlled and effective treatment option. Although not routinely included in current guidelines, orthodontic repositioning may be a valuable alternative in selected cases, particularly when rapid occlusal correction and restoration of jaw closure are required.

## Author Contributions

I. Middeljans contributed to the orthodontic treatment, data collection, and manuscript drafting. M. A. R. Kuijpers contributed to supervision of the orthodontic treatment, data collection, and manuscript drafting and revision. C. M. Suttorp contributed to the supervision of the orthodontic treatment, data collection, and manuscript drafting and revision.

## Funding

No funding was received for this manuscript.

## Ethics Statement

Ethical approval was not required for this case report. Written informed consent for the publication of clinical data and images was obtained from both the patient and his parents.

## Consent

A signed informed consent form has been obtained from both the patient and his parents. This form authorizes the publication of photographs and clinical data. The signed informed consent form has been uploaded to the Supporting Information section.

## Conflicts of Interest

The authors declare no conflicts of interest.

## Data Availability

All relevant data presented in this case report are included in the article. Additional information derived from patient records is not publicly available due to privacy restrictions.
